# On the Diseases and Injuries of Seamen

**Published:** 1853-09

**Authors:** G. R. B. Horner

**Affiliations:** Surgeon U. S. Navy, Philadelphia


MEDICAL EXAMINER,

AND

RECORD OF MEDICAL SCIENCE.

NEW SERIES.—NO. CV.—SEPTEMBER, 1853.

### ORIGINAL COMMUNICATIONS.

*On the Diseases and Injuries of Seamen.* By G. R. B. HORNER, M. D., Surgeon U. S. Navy, Philadelphia.

In our preceding remarks we have spoken of the means of obtaining healthy and capable persons for sea service, and of the best methods of keeping them efficient—many may be kept so—but in spite of all precautions, some cases of disease or injury will certainly occur among them, it matters not how small a crew may be. When it is large, on a long voyage, we must expect a corresponding increase in the number of disabled. It has appeared, then, that it might be useful for me to give an account of the diseases and injuries I have met with at sea, and of the curative means adopted, but chiefly of those which have been found the most efficacious, without undertaking to give a learned treatise, either on new or old remedies, and entering into a long disputation on the theory of the diseases, and the many modes of practice recommended since their first discovery. The reader likewise must not be disappointed, if he should find omitted many of the nice distinctions now made in their names, and which serve to create confusion and obscurity rather than to elucidate the nature of diseases. Pedantic terms may sound melodiously, and yet fail in sensè and imparting knowledge either respecting the manifold diseases to which the human race is liable, or the multitude of remedies used for their cure. For this latter reason a grand display of Latin prescriptions, setting forth long lists of medicines, puzzling the writer as well as reader to remember well, will be sedulously avoided—not only for this reason, but from my want of belief in the efficacy of making such heterogeneous compounds, and from my preferring simple remedies. After these preliminary remarks, I will observe, that although, as is well known, seamen are subject to the same diseases and injuries as landsmen, they are often modified by their peculiar mode of living. Seamen work, sleep, eat, drink, and dress differently from the former. At sea this happens from necessity; on shore from habit. The floating, peculiar structure of their ever-moving habitations, also produces changes in the disorders and injuries of seamen, and hence we find them varying frequently according to the class and state of their vessels, the general management of these, and the weather or climate where they are; yet, when any one disease becomes epidemic in a ship, at the same time that it commonly spreads with rapidity, from the crew being as it were imprisoned together, and it not being possible to perfectly separate the sick from the well, the type of the disease will be found very much alike. This will be rendered greater by their similarity of living, as respects food, drink, clothes, and other modifying causes. From the impossibility of flight, when an infectious or contagious complaint occurs in a vessel at sea, and far from port, all in her, except a few who may be protected from the disorder, know their liability to being attacked, and are not so susceptible of being seized with a panic as people on shore, and soon become accustomed to the danger, and resigned to whatever fate awaits them. Familiarity, too, with dangers from other causes blunts a sailor's sense of fear, and though his limited views into futurity should not make him a predestinarian, he suffers whatever evil befalls him with singular resignation, and meets death without fear. Hence I never knew this to kill a sailor, nor one to be accused by his physician of having died of fright. On the contrary, sailors bear disease with patience, meet death with philosophic complacence; and if they get well, pursue their erratic occupations and dissolute amusements with no apparent compunctions of conscience. Though they may read some of the numerous bibles distributed by societies in every vessel, and attend divine worship with great decorum, they are apt to do the first for pastime, and the last from the mechanical effects of discipline. The sacred books being put down, the service at an end, they become as reckless as ever, and think no more of what has been read or heard. When death at last is impending, they may take the medicines prescribed, from respect and obedience, but may care so little about it as to reject them, as I once saw done by an old sailor named Michael Leopold, dying of diarrhœa, caused by his being entrusted with a bottle of dissolved tartar emetic prescribed by the surgeon for a chronically enlarged testis. When he had been excessively prostrated, and the clammy sweat of dissolution was upon him, he cried out that "he was dying, and he was glad of it; take that brandy-toddy away," said he, "I will have none of it, for when I wanted it you would not give me any." Thus the old man died without further medicinal aid, and striking every person present with amazement at his refusing what no sailor was ever before known to reject in any shape whatever, for every one believes in the sovereign efficiency of grog. Internally they think it possessed of wonderfully healing powers—even greater than that of tar to any hurt, or of that panacea brown soap and sugar for an application to all boils and swellings, and external affections generally, whether the result of disease or injury. Some of the most ordinary external disorders to which seamen are liable are the former of every grade, from the smallest boil to the most aggravated phlegmon, partaking occasionally of a carbuncular character, involving all the tissues of the skin, and resting on the investing fasciæ of the muscles beneath. When this occurred the local inflammation occasioned great pain and febrile symptoms, the patient was unfit for duty, had to be taken on the sick list, and to undergo regular treatment.

The same had to be done in cases of less severity, and which were attended with only local symptoms, either from their disabling the affected parts, interfering with the proper use of the limbs, or it not being possible, while the patients were performing duty, to treat them conveniently. In cases of all kinds, these phlegmons would not be regarded as of local origin only, and in their treatment it was necessary to regulate the diet, forbid gross, crude, irritating ingesta, and to administer medicines internally to expel them, and restore the system to a healthy state. Saline cathartics were sufficient, usually. Severe cases required magnesia alone, or with epsom salts, or rhubarb, at the same time warm linseed poultices were applied, until suppuration had occurred, the phlegmons had came to a point, and then they were lanced so freely as not only to disgorge the cysts, turn out the hardened sloughs or cores in the centre, but to cause a copious effusion of blood from the parts surrounding the cysts. Afterwards, the poultices were renewed and reapplied until suppuration was complete, the parts had become soft and pale, and the pain subsided. The poultices were then left off, and some mild, soothing, astringent lotion used. A solution of the acetate of lead, in the proportion of a half drachm to a half pint of water, was commonly prescribed. Sometimes this lotion was introduced into the poultices to soothe the skin, and prevent a great extension of inflammation. Care was taken also to procure if possible flaxseed meal of good quality. In some places this cannot be obtained, and at Gibraltar the best that could be bought, instead of being soft, oily, and nearly white, was in cakes, hard, dry, dark, and like sawdust. Such flaxseed meal is said to be obtained from the seed after the oil has been entirely abstracted by heat and pressure, and is unfit for poultices. Better ones can be made of ship's biscuit, steeped in boiling water, and mingled with a little grease or oil, when sweet. If in port, fresh bread, made into a mass with milk, may be more convenient, and make a better substitute for a good linseed poultice: or one of mashed potatoes may be prescribed when bread is too dear, or cannot be had. For some phlegmons likewise an excellent poultice may be made by pouring boiling water on the inner bark of the red elm, after it has been cut or ground into small pieces. When thus prepared it becomes very pultaceous, soft, adhesive, and soothing.

Similar treatment to the above proved best in many other superficial affections, either of a local or general character, and arising from idiopathic or symptomatic causes. Local treatment was seldom alone adopted, and constitutional was never neglected when there was reason to believe the local disorders were mere signs of general ones, as was found in many eruptions, abscesses formed in the cellular substance of the axillæ, or about the lower part of the rectum, or beneath and at the root of the finger nails, constituting felons or whitlows. To these I have found seamen as liable as seamstresses, and have no doubt of their arising often in them from depraved digestion and bad diet, as well as local injuries and too great use of their fingers. During my late cruise, numerous cases of this kind happened at the same period that phlegmons were prevalent; and in one day, there were three admitted on the sick list for the former. A like treatment was followed, and as soon as the poultices applied had induced suppuration, free incisions were made into the cavities formed, to discharge their contents, prevent these from travelling beneath the nails, tendons and fasciæ, and producing caries of the bones, as I have witnessed. In one instance, those of a middle finger became so diseased, and caused such fungus in the joints and soft parts as to oblige me to amputate it at its junction with the metacarpal bone. From time to time similar abscesses were met with in the feet, beneath the fasciæ and tendons of the soles, and were also cured by following the above practice of poulticing first and then making free incisions. But one of the most remarkable abscesses ever seen by me, was occasioned by a chronic rheumatism in the left hip, buttock, and adjacent parts. The person affected was a gigantic seaman, a blacksmith by trade, of the name of Henry Fry, who got the above affection by exposure in the Brandywine at New York in 1830. After he had suffered considerably there and while she cruised, he returned to the United States, and became armorer of the John Adams early the next year. During her cruise in the Mediterranean, he suffered greatly from pain in the parts—principally in the left hip and buttock, extending along the course of the sciatic nerve; used friction with vol. liniment, and wore a Burgundy plaster on the parts. Other local remedies were used, and in the middle of January, 1833, he was taken on the sick list, being no longer able to perform his duties. The skin over and about the left trochanter major was of a dark brown color, and much indurated. He was unable to stand, and limped; but both of the inferior extremities were of natural size, and his general health was good. Treatment: Low diet, a poultice to the indurated parts, and a dose of calomel and jalap were directed. The next day, 20 leeches were applied, and the poultice continued. On the 18th, suppuration and ulceration had occurred; a fistulous orifice was formed over the lower part of the trochanter, and a probe was passed two inches and a half into the abscess. This increased rapidly, extended upwards and backwards, until it involved all the left buttock, and formed a number of other fistulæ above and below. One also broke out over the hip joint, and a scrofulous affection of it, as well as a lumbar abscess, were suspected. The purity of the pus discharged, its freedom from flocculi, the general absence of the marks indicative of a scrofulous diathesis, forbade such a conclusion; and the treatment was modified accordingly. It varied exceedingly, from the many turns the disease took. Sometimes, he was only locally affected, at others, he was feverish, and he was prescribed for accordingly, both local and general remedies. He took a number of blue pills, at intervals of one or more days; doses of Epsom salts and seidlitz powder, and rhubarb and magnesia in the morning; and had a solution of 5 grs. of the nitrate of silver in ℥i of water, injected into the abscess, to cause adhesion of its sides. A seton passed through two of the fistulæ, was used for the same purpose, and efficiently; the latter were repeatedly scarified—several incisions were made through the integuments, and finally, one almost a foot long was extended vertically down the buttock, to the back part of the trochanter, and in a line with the former. The gash was an enormous and frightful one—the parts divided were more than an inch thick; and had I known, before beginning, the extent of the abscess, I might have been deterred from it; but having made an incision of three or more inches, I did not reach the bottom of the abscess, and making another quite as long with a like result, I at last had to make a third one. Scraped lint was then laid in the abscess; the two lips of the wound were united with adhesive strips; a plaster of simple cerate and a compress put over it; some wine, warm rice water, a dose of laudanum, and friction with volatile liniment, were prescribed, to ease pain, and bring on reaction; for he had become faint, and was covered with a clammy sweat, although he had taken a dose of laudanum before the operation was begun.

After he had recovered from the immediate effects of it, the general treatment was renewed. This consisted chiefly of a diet drink made of sarsaparilla, sassafras and mezereon bark, and extract of liquorice macerated in water, and made into a decoction. But many other remedies were used, to counteract the untoward symptoms occurring from time to time—as fever, diarrhœa, swelling of the abdomen on the left side, and acute pain in different parts. Among these remedies were tartar emetic conjoined with the effervescing draught, acetate of ammonia, antimonial wine, tincture of opium, opium in substance, the sulphate of morphia endermically applied after blistering, and by enemata. On the 1st of June, three days after the operation, the dressings were removed, the wound was cleansed of a large quantity of clotted blood, and the bottom exposed. It was smooth, glossy and formd by the gluteal muscles chiefly, but it extended in various directions under the integuments, and had several fistulous outlets, one of which was on the posterior upper part of the thigh. On the 4th, the dressings were again removed, and I found that the lint had produced a healthy secretion of pus, and that granulations were springing up on all sides; but on the 9th, so much irritation was caused by the former, that the wound became inflamed, fever occurred, his pulse rose to 120 in the minute, and so much pain was occasioned in the hip joint, that the lint was taken out, and a neutral mixture, made of sup.-carb. of soda and vinegar, mingled with 50 drops of laudanum, was administered. The adhesive strips were renewed, as required; the nitrate of silver was applied also, to heal the lips of the wound; and after the fever had entirely subsided, to remove debility, he took the sulphate of quinia and diet drink. June 16th, the parietes of the abdomen anterior to the iliacus internus muscle, became tumid, and sore to the touch; continued afterward to swell, and were poulticed. Suppuration occurred; and on the 27th of August, I made an incision into the swelling, and discharged a large amount of pus. Becoming more prostrated in the mean time, he was given an infusion of cinchona ℥ij., pulv. gentian ʒss., pulv. zingiber ʒi., in lbs. ij. of boiling water. Lead water was used, to remove excoriation caused by the strips; a seton was kept in two of the fistulæ until their cavities were nearly closed; and by these means and similar ones, by the 12th of October, the wounds on the buttocks, as well as the fistulæ there, had perfectly healed, leaving a handsome cicatrix. Only one small fistula remained unhealed, and but a small quantity of pus issued from it; he had perfect use of the thigh; his general health had been restored, his strength, appetite and spirits had returned, and a speedy restoration to duty was anticipated.

This happy condition had been attained, although the ship had been cruising from one end of the Mediterranean to the other—had visited France, Greece, and Oporto—the whole summer conveying vessels through the Archipelago from Smyrna, and was then on her voyage homeward. Most unluckily, however, on the 2d of November, two days after she ran out of the straits of Gibraltar, he was seized with a catarrhal fever, ascribed to damp air on the Atlantic, and impelled before a strong Levanter, or east wind, which had driven the vessel out of the Straits against the rapid current making into them. A misty state of the atmosphere prevailed in this part of that ocean, which is about midway between the Straits and Madeira, and its ill effects I have frequently observed. To relieve him, a half drachm of laudanum and the same quantity of antimonial wine were given immediately, and the next day he took the last named article with a half ounce of spiritus mindereri every two hours. The sulphate of quinine was then discontinued. The symptoms still became more unfavorable, the fever continued, the fistula remaining in the left groin, increased to double its size, the skin extending from the former to the buttock became inflamed and erysipelatous, the cuticle peeled off, the buttock swelled, ulceration began in the cicatrices, after they had become livid and excoriated, and by the 11th, nearly all the newly formed parts had sloughed away, leaving a vast ulcer about three inches wide and about twelve long, or the whole length of the great incision. In the mean time his fever continued, the pulse was small and frequent, and his mind flighty. Nevertheless we did not despair, but continued the spiritus mindereri and antimonial wine, gave him hot marshmallow tea; bathed his feet in hot water; put a leech on each temple, and a lead water poultice to the inflamed parts. On the 7th, the fever having left him, the mixture was stopped, the poultice left off, an incision made into the upper part of the buttock, and a quantity of decomposed blood discharged. A plaster of mercurial ointment was then put on the buttock, the edges of the ulcer were brought together with adhesive plaster, and retained by a compress and belt. Delirium continuing, a sinapism was put to the nape of the neck, which relieved it; and to sustain strength, he was again given the diet drink and compound infusion of bark, with a fourth part of sherry wine in it. Great attention, as during the whole previous treatment, was paid to his regimen and general comfort. The mercurial ointment proving too stimulating, was diluted with three parts of simple cerate, and he soon began to recover. The sloughed parts healed rapidly, the fistula, by aid of frequent scarification and powdered cinchona, was filled up; and by continuing the above medicines as long as needed, and giving the sulphate of quinine, he still more improved. He also took a blue pill every other night, a dose of powdered rhubarb and calcined magnesia on the morning after for two days; and by the middle of the following January, the hip and buttocks were again perfectly healed.

Finally, by the 3d of February, notwithstanding the ship had been at sea nearly the whole time,—had visited Mogadore, gone to Madeira and Teneriffe, passed through the calm, hot latitudes between that island and Liberia, spent several days at Monrovia, thence crossed over to the West Indies, stopped a week at Martinique, and for a fortnight had been blown off the coast of the United States by a cracking northwester, which raised a tremendous sea, and greatly rocked her,—then all parts entirely healed, he walked without help, and was then transferred to the naval hospital at Norfolk. From that he was soon discharged, well, applied some time afterwards, at the Philadelphia rendezvous, to reship, and being perfectly sound, was taken into service again, to make the air resound with his sledge hammer and anvil. Though not lame, as his progenitor, Vulcan, he strongly reminded us of him, as his muscular arm and huge body heaved up and down, and his heavy blows were laid on the red hot iron. But I regret to add, that this valuable seaman died some years afterwards, on board one of our national vessels, of dysentery, contracted during his cruise in the East Indies.

Another instance of abscess of the left buttock, had occurred in the same vessel, and in the person of the ship's cook, a slender, decrepid man of 45 years of age, who also had a cataract in the left eye and amaurosis of the right one; besides, was dispeptic and diabetic. As in the preceding case, the disease was preceded by symptoms of sciatica, pain in the hip joint, and swelling over the left sacro-sciatic notch. He was taken under treatment, likewise, during the winter, was cupped, leeched several times, blistered, poulticed, took blue mass repeatedly, magnesia, &c. to work it off; had the blistered surface sprinkled nightly with the acetate of morphia, and was given, besides the tonics mentioned, an infusion of quassia. For the benefit of his eyes, a seton was introduced and worn for weeks in the nape of his neck. But five months after his admission on the list, and during the following May, the abscess had perfectly formed, and being about to ulcerate through the skin, was opened by a valvular incision. About five ounces of flocculent, straw-colored pus, were let out. It continued to flow for more than three weeks; he became prostrated and hectical; and as the ship was bound on a long cruise into the Atlantic, he was transferred to the flag ship Brandywine. Of what became of him afterwards I have never heard, and cannot give the result of his complaint, but surmise it was a fatal one.

The following case of abscess of a different kind, was sent to the John Adams from the Constellation frigate, to be carried home. The person was J. D., a young, handsome, delicate marine, who was said to have an affection of the urinary organs, ascribed to a fall down a ladder. He had undergone little treatment, and, according to his own account, taken only one dose of medicine, a large one of calomel. Being of scrofulous habit, this had salivated him, and caused soreness of the mouth, with ulceration of the gums. He was then weak and hectical: and, a day or two after he came on board the ship, was observed to have a small swelling on the left side of the nose, and its bridge. He stated, he had had the former broken when he was a boy, and the venereal disease eight years before. There was no other evidence of this having any thing to do with his ill health, and he was treated correspondingly, and for mercurialism.

An incision was made into the swelling—a half ounce of pus discharged; and the cavity being probed, the left nasal bone was found rough and carious. He was put on a plain, nutritious diet: took magnesia, to correct acidity of the stomach; had the lunar caustic applied to the fungus, which sprung out of the wound made on the nose. He took the sulphate of quinine, and two diet drinks, of which the following was the principal. It consisted of sarsaparilla, ℥viij., lignum guaiaci, ʒij., sassafras, ℥iv., mezereon, ʒij., honey, ℥viij., and water, two gallons, which was boiled down after maceration to two gallons. This decoction was cooled, strained, and taken as required. In the second case, the guaiacum and mezereon, which were too acrid and stimulating, were left out, and senna leaves added. After this decoction had been boiled down as the other, it was sweetened with loaf sugar, and used in the manner as above. Three weeks after he was taken on board, the alveolar processes of the upper jaw were found carious, and the nasal process of the superior maxillary bone, as well as the nasal, eventually became affected. In the meanwhile the ship had got to Liberia, he had been more prostrated by the heat of the climate, and was prescribed bathing in cold sea water. By this treatment, he recruited, reached the United States safely, and some time subsequently recovered his health, with the loss of the bridge of his nose. This case is, I consider, a very striking one of the positively poisonous effects of mercury, and it proves the great impropriety of giving it without regard to the constitution of patients. It moreover appears, that it is given wrongfully in other than syphilitic cases, and that its use should be avoided when other medicines can be as well employed without such consequences. To show how usefully the preparations of mercury may be used, and yet prove injurious, I will give the account of another case, that of Samuel Mahoney, a young sailor of 22 years, tall, slender, and disposed to disease of the lungs. He had been treated 18 months previously for pleuro-pneumonia, and cured by means of V. S., nitrous powders of the common kind, containing calomel, thirty-six leeches to the chest, the spiritus mindereri, and tart. emet. blisters, the sulphate of morphia, and demulcent drinks. He was readmitted for a tumor on the posterior face of the right olecranon process, as large as a hen's egg, and resembling a wen. He stated, that three months before he had fallen on the elbow, and the tumor had formed since then. As it was increasing rapidly, it was cut off by an elliptical incision, and was discovered to have been formed of a thick sac, containing a half ounce of yellowish serum, a fibrous mass adhering to the internal surface by tendinous bands. The base of the tumor adhered so closely to the bone that it could not be perfectly detached. After the incision, the edges of the wound were brought together with adhesive strips, a compress of lint was applied over them, a pasteboard splint was put in front of the joint, and a roller passed around the fore arm and it, to prevent flexion and the protrusion of the naked olecranon through the wound. These things done, he took a dose of Epsom salts and magnesia; and these medicines having worked too actively, and with the bleeding caused too much debility, he was given a dose of laudanum and tinc. of lavender. The dressing remained undisturbed for several days, and was then removed. Every attention was paid him; he was kept on low diet, and at rest, and yet the wound became unhealthy. Granulations sprung up, and it filled; became fungous; projected a half inch above the lips; a number of large pustules formed on the adjacent skin; a solution of sugar of lead was used as a wash for these, and the lunar caustic applied to the fungus time after time, and as often as the dressing was removed. Notwithstanding, the fungus continued to grow, and I determined to try constitutional with local treatment. After the operations had been done for more than a month, I prescribed blue mass, five grains every other night, and powdered rhubarb, ℈j, with magnesia, ʒj., the next morning, and the caustic was applied occasionally. This treatment had a most happy effect. The fungus quickly sank down, the edges of the wound approximated, and, by the 10th of January, three weeks before the ship arrived at home, a perfect cicatrix had formed, and the elbow was well. This cure strongly illustrated the utility of mercury; but to show what injury it at the same time did, I will state that, on the 17th of the previous month, without any reason to suspect venereal taint, the right inguinal glands began to swell; a bubo formed and suppurated: twenty days afterwards a second one formed in the same part; and five days subsequently a third one appeared in the left groin, between the scrotum and thigh. The latter bubo was indolent; the two first, on being poulticed, formed a single large abscess, and the last of the two discharged its contents through the incision of the first one. All of them were obstinate, and resisted local and general remedies. The nitrate of silver, Turner's cerate, cathartics, blue mass, and Lisbon diet drinks were of little service, and, in February, he was sent to the naval hospital of Norfolk, the buboes having been still unhealed, although his elbow remained sound. Hence, I was well pleased with the action of the blue mass in this respect, but was displeased at the palpable effect it had had in developing a scrofulous diathesis. Had the patient had chancres, or have been known to have been exposed to syphilis, the buboes might have been attributed to it; but neither was the fact; and his confinement to the ship or her boats for at least a year, rendered it impossible for him to have been infected within that period, as women were excluded from her.

A case of not less importance, if not of more, was that of a seaman transferred to the John Adams, at Athens, in Sept. 1833, from the flag-ship United States, which the year before had arrived in the Mediterranean. This case was also attended with enlargement of the inguinal glands, but he had in addition an induration of some of the lymphatic ones of the neck; one of the parotids was swelled, and there was extensive ulceration of the fauces, which had destroyed the right tonsil, and was attended with inflammation and tumefaction. He had been affected for nearly seven weeks, and nearly all his teeth were decayed. The molars had suffered most, pus filled the hollows, the gums were black, and a fistulous ulcer had penetrated the right side of the velum pendulum palati. His voice was hoarse and guttural, and deglutition difficult; the edges of the ulcers were thickened and inflamed; and, as he had had no venereal affection, was thin, pale, and had stronger marks of being of a scrofulous diathesis, his affection was considered to be of that nature; though, so far as I could learn, he had been treated only for an ordinary ulcerated throat, and had been using a number of gargles; among which there were two of the sulphate of copper and nitrate of silver.

Agreeably to my opinion of the scrofulous nature of his disease, an ointment of iodine, ʒj., basilicon, ℥j., was spread on a rag, and directed to be applied over the right parotid gland twice a day. He was also given a solution of iodine and hydriodide of potash, in distilled water, and in the dose of twenty drops, four times a day. The dose was gradually increased to forty drops, and a strong solution of the nitrate of silver was applied twice a day to the ulcers by means of a mop.

In spite of this treatment the ulceration continued, penetrating the velum in another place, and then united the two parts together, so that it fell to the left side, and rested on the tongue. A sulcus was formed in the roof the mouth, and the palatine bones seemed to have been destroyed by caries. In the course of two weeks, however, the parts had improved, and the ulcer on the right side of the fauces had healed, though the velum on that side was nearly obliterated, and the uvula was considerably inclined to the other. The disease, however, continued in other parts, the fistula was detected in the right anterior half arch five weeks after he came under my care, and the solution of the nitrate of silver was reapplied by means of a hair-brush. This fistula remaining stationary, was then laid open with a scalpel, and bleeding very profusely, a solution of alum, in the tinct. of kino was successfully applied as a styptic; a diet drink of a similar kind to those mentioned was given. He took blue mass grs. x. every other night, the caustic was reapplied as required, the ulcers were scarified with a lancet repeatedly, and yet a month later the fauces were more inflamed, and so continued until in using the wash the half of what appeared to be the body of a dead bread worm was extracted from the ulcer in the velum. In a short time subsequently the throat became much less inflamed, the internal remedies were continued, and a gurgle of sulph. of zinc and sage tea used until it lost its good effect, and was then left off for the caustic in solution and substance. To remove costiveness several doses of cream tartar and sulphur in molases were given. Under this treatment, varied as required by the condition of the patient, his health was restored, the perforations in the palate closed, and the other ulcerated parts nearly healed by the time of his arrival in the United States. His transfer to the hospital prevented me from effecting a perfect cure, and this was the greater subject of regret as he had been under my care almost five months.

